# The Burden of Drug Abuse in Nigeria: A Scoping Review of Epidemiological Studies and Drug Laws

**DOI:** 10.3389/phrs.2021.1603960

**Published:** 2021-01-29

**Authors:** Abubakar Ibrahim Jatau, Abubakar Sha’aban, Kabiru Abubakar Gulma, Zayyanu Shitu, Garba Mohammed Khalid, Abubakar Isa, Abubakar S. Wada, Mohammed Mustapha

**Affiliations:** ^1^ School of Pharmacy and Pharmacology, University of Tasmania, Hobart, TAS, Australia; ^2^ School of Pharmaceutical Sciences, Universiti Sains Malaysia, Penang, Malaysia; ^3^ Department of Clinical Pharmacy and Pharmacy Practice, Faculty of Pharmaceutical Sciences, Ahmadu Bello University, Zaria, Nigeria; ^4^ School of Global Health and Bioethics, Euclid University, The Gambia; ^5^ Hospital Services Management Board, Ministry of health, Gusau, Nigeria; ^6^ Department of Pharmaceutical Sciences, Università Degli Studi di Milano, Milano, Italy; ^7^ Malaria Consortium Nigeria, Dutse, Nigeria; ^8^ Department of Pharmacology and Therapeutics, Faculty of Pharmaceutical Sciences, Bayero University Kano, Kano, Nigeria

**Keywords:** drug abuse, cocaine, Nigeria, codeine, public health, reviews

## Abstract

**Background:** The burden of drug abuse is becoming a public health concern in Nigeria. Preventive measures should include identifying the root causes of the burden for targeted intervention. We, therefore, aim to conduct a scoping review of the literature to summarize the findings of epidemiological studies on drug abuse and provisions of drug laws in Nigeria. The review also provides appropriate recommendations as interventions for prevention.

**Methods:** We conducted a systematic search of the literature on PubMed to identify information on drug abuse and drug laws in Nigeria from the inception of the database to March 2020. Additional information was retrieved from Google Scholar, a manual search of included articles, discussion with experts on the subject matter, and gray literature. Study selection was performed using the Preferred Reporting Items for Systematic Reviews and Meta-Analyses (PRISMA) statements. Information from gray literature was assessed for quality and accuracy using the AACODS checklist (authority, accuracy, coverage, objectively, date, significance).

**Results:** The systematic search of the literature generated 253 studies. Nine articles were obtained from other sources. After the selection process, 23 eligible studies were included for review. A prevalence of 20–40% and 20.9% of drug abuse was reported among students and youths, respectively. Commonly abused drugs include cannabis, cocaine, amphetamine, heroin, diazepam, codeine, cough syrup and tramadol. Sources where abusers obtained drugs, were pharmacies/patent medicine shops, open drug markets, drug hawkers, fellow drug abusers, friends, and drug pushers. Drug abuse was common among undergraduates and secondary school students, youths, commercial bus drivers, farmers, and sex workers. Reason for use included to increase physical performance, stress and to derive pleasure. Poor socioeconomic factors and low educational background were the common risk factors associated with drug abuse. We identified several drug laws and policies that were established under government agencies such as the National Drug Law Enforcement Agency (NDLEA), National Agency for Foods and Drugs Administration and Control (NAFDAC), Pharmacists Council of Nigeria (PCN) and a Presidential Advisory Committee.

**Conclusion:** Findings from epidemiological studies on drug abuse in Nigeria has demonstrated that the burden of drug abuse is still high despite the existing drug laws, policies, and strategies for prevention. Measures to reduce the burden should involve the community, government, and religious bodies. Preventive measures should target the youths, the students, identified sources of the drugs, reasons and risk factors associated with drug abuse in Nigeria.

## Introduction

Drug abuse is emerging as a global public health issue. The recent world drug report-2019 of the United Nations Office on Drugs and Crime (UNODC) estimated that 271 million (5.5%) of the global population (aged between 15 and 64 years), had used drugs in the previous year [1]. Also, it has been projected that 35 million individuals will be experiencing drug use disorders [1]. Further, the Global Burden of disease Study 2017 estimated that, in 2017, there were 585,000 deaths due to drug use, globally [1]. The burden of drug abuse (usage, abuse, and trafficking) has also been related to the four areas of international concern, *viz.* organized crime, illicit financial flows, corruption, and terrorism/insurgency [2]. Therefore, global interventions for preventing drug abuse including its impact on health, governance, and security, requires a widespread understanding of the prevalence, frequently implicated drugs, commonly involved population, sources of the drugs and risk factors associated with the drug abuse.

In Nigeria, the burden of drug abuse is on the rise and becoming a public health concern. Nigeria, which is the most populous country in Africa, has developed a reputation as a center for drug trafficking and usage mostly among the youth population [5, 6]. According to the 2018 UNODC report “Drug use in Nigeria”—The first large-scale, nationwide national drug use survey in Nigeria, one in seven persons (aged 15–64 years) had used a drug in the past year [3]. Also, one in five individuals who had used drug in the past year is suffering from drug-related disorders [3]. Drug abuse has been a cause of many criminal offences such as theft, burglary, sex work, and shoplifting [3].

Nigeria is an enormously diverse country with over 400 ethnicities and many religious groups [9]. Drug abuse is therefore viewed within a broader context in Nigeria, due to its multicultural nature. For instance, most societies do not consider the use of some drugs which do not produce overt behavioral changes as drug abuse. However, despite this multicultural nature of the Nigerian population, there is a consistent outcry from both the public, police, preachers, health professionals, teachers, regulatory agencies and parents on the growing burden of drug abuse (abuse of drugs which affect behavior) in the country. The recent call was that of the President of the Pharmaceutical Society of Nigeria [4].

Efforts to prevent the growing burden of drug abuse in Nigeria involve the identification of evidence-based information on the extent of the problem, from epidemiological studies. To date, most of the information on drug abuse in Nigeria is reported by the media (print, electronic and online). However, scientific evidence from epidemiological studies has started emerging in recent years. Although there were attempts in the past to summarize such studies in the form of a narrative review, such reviews were limited with lack of systematic search of the literature. Also, such studies were published in 1982 [5], 1991 [6], and 1992 [7]. There is, therefore, a need for recent summarized data on drug abuse in Nigeria. We, therefore, aim to conduct a scoping review of the literature to summarize the findings of epidemiological studies on drug abuse and provisions of drug laws in Nigeria. In this review, we attempt to summarize the results from various studies regarding the prevalence of drug abuse, commonly abused drugs, sources of the drugs, group of people frequently involved, the reason for drug abuse, risk factors, extant policies and laws, and to recommend intervention measures for prevention.

## Methods

### Literature Search

In the literature search, we have not restricted our sources of information to any specific period. A systematic search of the literature regarding drug abuse in Nigeria was conducted using PubMed from the inception of the database to March 13, 2020. A search strategy using the following terms both as medical sub-heading (MeSH) and free text as title and abstract (tiab) was developed. The search terms used included: “abuse drug” [MeSH], “abuse drug” [tiab], “illicit drug use” [tiab], “drug, illicit” [MeSH], “psychoactive drugs” [MeSH], Nigeria [tiab]. Relevant studies were also identified manually from the reference lists of the included articles and discussion with experts on the subject matter. Additional information was also retrieved from Google Scholar using the following search expression “Drug abuse, illicit drug abuse, psychotropic abuse in Nigeria.” Based on previous recommendations, only the first 200 search results from the Google scholar search were considered for inclusion [8]. The search strategies employed in the systematic search of the literature in PubMed and Google Scholar is provided in Appendix 1. Due to limited research on drug abuse in Nigeria, gray literature related to drug abuse in Nigeria were identified in Google search, web pages of drug regulatory agencies in Nigeria, and the UNODC. Information from gray literature was evaluated for trustworthiness and relevance based on AACODS (Authority, Accuracy, Coverage, Date, Significance) checklist [9].

### Study Selection

**FIGURE 1:**
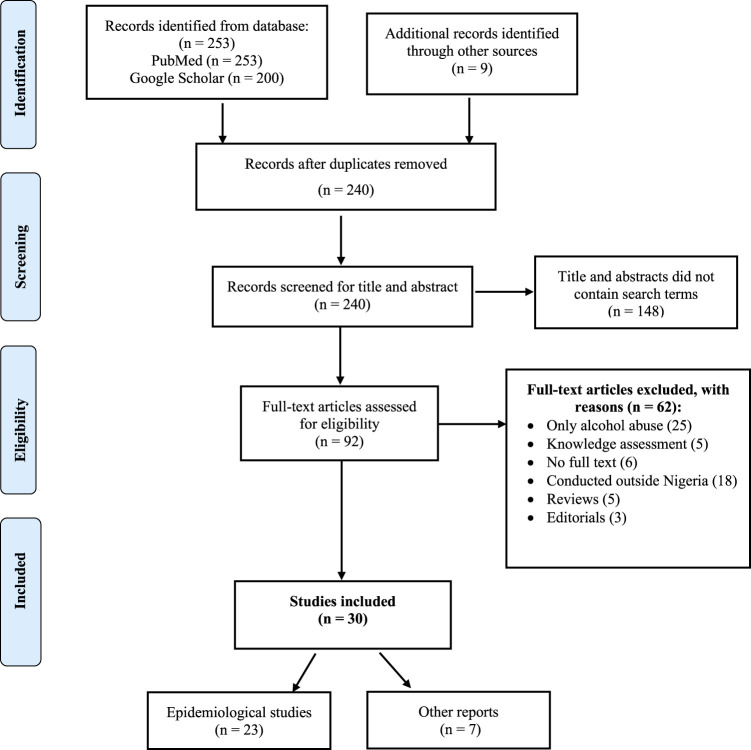
PRISMA Flowchart for the study selection process.

Studies or reports were included in this review based on the following criteria: reporting prevalence/incidence of drug abuse in Nigeria; conducted among different populations in the community, government policies and interventions on drug abuse; and drug laws in Nigeria. Studies or reports were excluded from this review if they focused on alcohol abuse only. Disagreements between authors regarding study selection were resolved through discussion until consensus was reached. Figure 1 demonstrates the study selection process.

### Data Extraction

Information extracted from the included studies included: study authors, population/sample size, settings, prevalence, commonly abused drugs, sources, reasons, and factors associated with the drug abuse.

### Operational Definition

There was no universal definition of the term “Drug abuse” in the literature. In this review, the definition of drug abuse by Benjamin and Chidi (2014) was adopted and modified [10]. “the use of a drug that is not generally accepted on medical ground, i.e. continuous or occasional use of drugs that would cause overt behavioral change by the individual either of his own choice or under a feeling of compulsion, to achieve his wellbeing or what he conceives as of his own wellbeing” [10].

## Results and Discussion

The initial search PubMed and Google Scholar generated 53 articles and 200 records, respectively. One study was identified following discussion with an expert, two from a manual search of included studies and six articles from Google search. The screening for duplicates excluded twenty-two articles. One hundred and forty-eight articles were removed after screening for title and abstracts, and 62 studies did not meet the eligibility criteria and were excluded. A total of 30 studies were included in this review; 23 epidemiological studies [3, 11–21, 23–32], one thesis inclusive [22], five reports from the websites of drug-related agencies [38, 41, 43, 44] and UNODC [2], and two articles from newspapers[40, 45]. Figure 1 illustrates the study selection process.

Nine of the included epidemiological studies were conducted among secondary school students [12, 16, 21–24, 27, 30, 31], five among drug abusers [15, 18, 25, 29, 32], four among undergraduate students [17, 20, 26, 28], three among members of different populations in the community [3, 11, 14], two among commercial vehicle drivers [13, 19]. Table 1 shows the characteristics of the included studies.

**TABLE 1:** Summary of the included epidemiological studies.

Author/year	Population	Setting	Prevalence/commonly abused drugs	Sources of drugs	Reasons	Risk factors
United nations office on drugs and crime. 2018 [3]	38, 850 households	Community centers, houses in the 36 states and FCT	Prevalence (14.4%)	Not reported	Not reported	Male gender, age 25–39 years, sex worker
	9,344 drug users					
	2,787 key informants					
	National survey (36 states and FCT)		Cannabis (10.8%), opioids (tramadol, codeine, or morphine) (4.7%), cough syrup-containing codeine and dextromethorphan (2.4%), tranquilizers (0.5%), amphetamines (0.2%), cocaine (0.1%), heroin (0.1%)			
Gobir et al., 2017 [11]	359 youths from the general population	Rural communities in Sokoto state	Prevalence (10%)	Not reported	Increase physical performance	Being a farmer
	Aged 15–35 years		Tramadol (52.8%), cannabis			
Erah 2017 [12]	187 secondary school students	Three secondary schools	Prevalence (20.9%)	Not reported	Not reported	Male gender
	Mean age, 16 years	A state in the south-south region	Cannabis (3.2%), caffeine (5.9%), codeine (5.3%), nicotine (2.1%) and cocaine (1.6%)			
Yunusa et al., 2017 [13]	196 commercial bus drivers	Eight motor parks in Kano metropolis, Kano state	Prevalence (81.1%)	Not reported	Desires to relax/sleep (84.8%), physical performance (48%), stress (81%), anxiety (66.5%), pleasure (72%)	Male gender, lower education level, lower monthly income
	Mean age, 32.3 years		Tramadol (80.6%), herbal tea (*Gadagi*) (78.1%)			
Namadi 2016 [14]	480 adolescents	Kano metropolis, Kano state	Cocaine (0.6%), heroin (0.6%), codeine (22.7%), cough syrups (26.1%), cannabis (22.7%)	Not reported	Motivation, stress, build up self-esteem, performance, poverty, unemployment, frustration, hedonism, reduce fear, relaxation	Parental deprivation (broken home), stress, depression, self-esteem, unemployment, poverty
	25 years and below					
Dankani 2012 [15]	487 of identified drug abusers	Schools, joints, NDLEA offices and psychiatric hospitals in Kano, Katsina, Kebbi, Sokoto and Zamfara state	Codeine-containing cough syrups, cocaine (NR), heroin (NR), ephedrine (NR), caffeine (NR), barbiturates (NR), amphetamine (NR)	Chemists/pharmacies (33%), drug wholesalers (23%), fellow drug abuser (8%)	Experiment (9%), boredom (6%), depression/anxiety (17%)	Male gender, age 21–30 years, peer-group influence, parental deprivation (broken home)
Famuwiya et al., 2011 [16]	4,286 secondary school students	Ten secondary schools in Lagos state	Barbiturates (17.5%), minor tranquilisers (16.4%), fencamfamine (Reactivan®) (7.9%), Methaqualone (Mandrax®) (7.6%), cough medicines (33.2%), heroin (4.1%) dexamphetamine (4.5%), cocaine (4.8%), cannabis (4.7%)	Not reported	Easy access	Male
	Mean age, 15.2 years					
Essien 2010 [17]	200 undergraduate students	Federal university of technology, Minna, Niger state	Cocaine (10%), heroine (1%), codeine (3%), cannabis (11%)	Not reported	Stress (12.5%), self-esteem (15%), performance (6.5%), euphoria (13.5%), poverty and unemployment (8.5%), pleasure (3.5%), reduce fear (12.5%), relaxation (6.5%)	Age 15–35 years, polygamous family
Adamson et al., 2010 [18]	All psychiatric cases in periods 1992–1997 (109 cases) and 2002–2007 (105 cases)	The drug addiction treatment, education and research Unit (DATER) of the neuropsychiatric Hospital, Aro, Abeokuta, Ogun state	Cocaine (44%), heroin/opiates (22%), cannabis (53.5%)	Not reported	Not reported	Unemployment, students, male, last born, having a parent who is drug users
	Mean age 17 years					
Makanjuola et al., 2007 [19]	69 Licensed commercial long-distance vehicle drivers	Four motor parks in Ilorin, Kwara state	Anabolic steroids (27.5%), sedatives (10.1%), cannabis (0.6%)	Not reported	Keep awake, experiment, increase performance	Being religious (protective), male gender
	Mean age, 44 years					
Makanjuola et al., 2007 [20]	961 undergraduate students	University of Ilorin, Kwara state	Sedatives (7.3%), anabolic steroids (0.4%)	Not reported	Not reported	Male gender, religion (protective)
	Mean age, 22.4 years					
Abdulkarim et al., 2005 [21]	1,200 secondary students	Six secondary schools in Ilorin, Kwara state	Prevalence of 40.1%	Not reported	Not reported	Being a cigarette smoker
	Aged 10–19 years		Amphetamine and ephedrine (6%), heroin (4%), cocaine (3.6%), cannabis (34%)			
Edafiadhe 2005 [22]	750 secondary school students	Nine secondary schools in Edo state	Prevalence (32%)	Friends (41.2%), drug pusher (3.0%), family (1.6%), physician (8.3%), other health practitioners (3.0%)	Sociability and acceptability, experiment	Male gender, peer-group influence
	Mean age, 17 years		Cocaine (1.6%), amphetamine (8%), hallucinogen (1.6%), tranquilizers (1.8%), sedatives (7.8%), heroin (4.8%), cannabis (6.4%)			
Lawoyin et al., 2005 [23]	394 secondary school students	Three secondary schools in oyo state	Acetylsalicylic acid and caffeine (Alabukun®), (NR)	Not reported	Not reported	The social relationship of the user (close friend and primary caretaker), male gender
	Adolescents and youths					
Eneh and stanley 2004 [24]	1,049 secondary school students	Four secondary schools in Rivers state	Cannabis (26%), butazolidine (39.3%), codeine and dexamphetamine (Pemoline® (28%), diazepam (24%)	Not reported	Not reported	Not reported
	Adolescents					
Ohaeri and odejide 1993 [25]	A retrospective study of records of all (10,396) patients admitted in 1989	All (14) psychiatric care facilities in Nigeria	Cannabis (NR), cocaine (NR), pethidine (NR), amphetamine (NR), a mixture of aspirin (NR), codeine and dexamphetamine (Pemoline ®) (NR), barks of unidentified trees (NR)	Patent medicine stores, drug hawkers, hawkers of traditional herbal preparations	Keep awake or to have increased energy for work, easy access	Family background of lower socioeconomic status, unemployment, male gender
Adelekan et al., 1992 [26]	636 undergraduate students	University of Ilorin, Kwara state	Cannabis (8%), hypnotics (diazepam, chlordiazepoxide) (17.9%), heroine/morphine (0.6%), cocaine (0.6%)	Not reported	Experiment/curiosity	Not reported
	Mean age, 23 years					
Akpala and Bolaji 1991 [27]	306 secondary school students	Three secondary schools in sokoto state	Prevalence of 41%	Friends (61%), relatives (10%), parents (5%), teachers (3%)	To relieve worry and anxiety (17.6%), to feel happy (12%), facilitate reading (7%), stay awake at night (8.8%), induce sleep (14.4%), enjoyment of social activities (20%), fun (8.8%)	Male students, age 25–29
			Cannabis (12%), diazepam (18%), amphetamine (9%), methadone and diphenhydramine (Mandrax®) (2%)			
Ihezue 1988 [28]	775 undergraduate students	University of Nigeria, Enugu state	Cannabis (11%), tranquilisers (13.4%), narcotics (codeine) (8.2%), sedatives (3.6%), stimulants (1.1%)	Not reported	Not reported	Male gender, poor academic performance, peer group influence, a family background of lower socioeconomic status, parental deprivation (broken home)
Ahmed M.H 1986 [29]	A retrospective study of 367 new cases of drug abuse	Department of Psychiatry, Ahmadu Bello university teaching hospital, Kaduna state	Amphetamine (19%), Methaqualone (Mandrax®) and phenobarbitone (25%), cannabis (54%)	Not reported	Not reported	Age 15–30 years, male, single
Nevadomsky J. 1982 [30]	500 secondary school students	Six secondary schools in Delta state	Cannabis (47%), caffeine (Proplus®) (15%), Methadone and diphenhydramine (Mandrax®) (13%), Reactivan (28%), chlordiazepoxide or diazepam (34.2%)	Not reported	Experiment	Peer group influence
	Mean age, 17 years					
Nevadomsky J. 1981 [31]	1, 500 secondary school students	18 secondary schools in Bendel state (the current Edo and Delta states)	Cannabis (0.6%), caffeine (Proplus®) (0.2%), Methadone and diphenhydramine (Mandrax®) (0.7%), chlordiazepoxide and diazepam (1.9%), LSD (0.06%)	Underground agents (57%), chemists (45%), home (33%), open markets (17%)	Experiment/curiosity, boldness, to feel happy, sleep well, stay awake, academic pressure, stay calm, loneliness, sports	Parental deprivation (broken home), peer group influence
Oviasu 1976 [32]	Review of 491 cases of drug abuse	Uselu nervous Diseases Clinic, Edo state	Caffeine (Proplus®) (0.2%), amphetamine (3.1%)	Pharmacy, patent medicine shops, drug hawkers	To improve intellectual and physical performance, to stay awake	Age under 20 years, male gender, secondary school students, polygamous family background

FCT, Federal Capital Territory; LSD, Lysergic acid diethylamide; NR, Percentage not reported in the study.

### Prevalence of Drug Abuse in Nigeria

Of the 23 epidemiological studies, only seven reported an overall prevalence of drug abuse among the study sample [3, 11-13, 21, 22, 27]. Given the heterogenic nature of the studies, determination of the pool prevalence of drug abuse in Nigeria may not be possible. Four of the studies were conducted among secondary school students and reported a prevalence between 20 and 40% [12, 21, 22, 27]. A prevalence of 14.4% was reported among members of the general public (all ages), 20.9% among youths in the community [11], and 81.1% among commercial bus drivers [13].

### Commonly Abused Drugs in Nigeria

The most frequently implicated drugs, consistently reported by the majority of the studies were; cannabis [3, 12, 14, 16-19, 21, 22, 24-31], codeine [3, 12, 14, 15, 17, 24, 25, 28], amphetamine/dexamphetamine [3, 14-17, 21, 22, 24, 25, 27, 29, 32], heroin [3, 14, 16–18, 21, 22, 26, 27], cocaine [12, 14, 15, 17, 18, 21, 22, 25], diazepam [26, 27, 30–32], and cough syrup [3, 14–16], Reactivan (fencamfamine) [16, 31], Mandrax [27, 29–31], and tramadol [3, 11, 13].

Some drugs were frequently reported by studies published in the early 80s [29–32]. Proplus (caffeine 50 mg) was reported by three papers published in 1982 [30–32], and Madrax (Methadone and diphenhydramine) in studies published between 1981 and 2011 [27, 29–31]. The absence of these drugs in recent studies may be related to the decline in their availability in Nigeria.

Cannabis was the most abused drug reported across the different study populations. The prevalence of cannabis abuse among members of the general public was 10.8% [3] and 22.7% among adolescents of 25 years and younger [14]. The frequency of abuse among secondary school students was between 0.6 and 34%, with a pooled prevalence of 12.5% [12, 16, 21, 22, 24, 31]. The abuse of cannabis among undergraduate students was also common, with a prevalence of 8–11% [17, 26, 28].

The frequency of cocaine abuse ranges from 1.6 to 4.8% among secondary school students [12, 16, 21, 22], 0.6–10% among undergraduate students [26, 28, 33] and 0.1–0.6% among members of the general public [3, 14]. The widespread use of cocaine in Nigeria may be related to easy access due to increased trafficking of drugs despite the existing legal control measures [22, 34].

Codeine was the third most frequently reported drug of abuse from the included studies. The prevalence of abuse in the general public (all ages) was 2.4% [3], and 22.7% among adolescent [14]. A prevalence of 3–8.2% [17, 28], and between 5.3 and 28% [12, 24] was recorded among undergraduate students and secondary school students respectively. Table 1 indicates the frequently abused drugs. The high rates of drug abuse among the younger persons could reflect the easy accessibility of these drugs, peer group influence and possibly lack of effective counseling programs in secondary schools and universities.

### Sources Where Drug Abusers Obtained Drugs

Identifying the sources where drug abusers obtained drugs is essential in preventing drug abuse in Nigeria. Interventions to block the supply of these drugs from identified sources could reduce the increasing prevalence of drug abuse. Only five studies reported the sources of the drugs being abused. The common sources included: pharmacies/patent medicine shops (23–33%) [15, 25, 31, 32], open markets (17%) [31], drug hawkers [25, 32], hawkers of traditional herbal preparations [25], fellow drug abusers (8%) [15], underground agents (57%) [31], family members (1.6–33%) [22, 27, 31], friends (up to 61%) [22, 27], teachers (3%) [22, 27], physician (8.3%) [22], other health practitioners (3.0%) [22].

### Reasons for Drug Abuse in Nigeria

Determination of the reasons why people indulged in drug abuse may guide the development and implementation of targeted interventions for reducing the burden of drug abuse in Nigeria. The eleven studies that reported the reasons for drug abuse gave several reasons. The commonly reported reasons included the following: to increase physical performance [11, 13, 14, 17, 19, 30, 32], to drive pleasure [13, 14, 17, 27, 30], desire to relax/sleep [13, 14, 17, 27, 30], experiment/curiosity [15, 19, 26, 30, 31], to keep awake [19, 25, 27, 30, 32], to relieve stress [13, 14, 17], to relieve anxiety [13, 15, 27], unemployment [14, 17], frustration [14, 15], and easy access [16, 25].

Exterior or curiosity motives, often in the form of extreme explorative curiosity to experience ‘the unknown’ about drugs, motivate individuals into drug use and subsequent drug misuse and abuse. The first experience in drug abuse produces a state of arousal in the form of extreme happiness and pleasure, which in turn motivates users to continue [34]. With the high poverty rate of about 50% of people living in extreme poverty in Nigeria [35], and the rising rate of unemployment (23.1%) [36], indicate how challenging the socioeconomic condition could be for many Nigerians. These conditions could predispose people to engage in drug abuse to work harder to earn a living or to ward off the stress and frustration of daily living in hardship.

### Risk Factors Associated With Drug Abuse

Nineteen of the included studies examined factors that could raise people’s likelihood of drug abuse [3, 11–23, 25, 27–32]. Among the frequently reported factors included being a male gender [3, 12, 13, 15, 16, 18-20, 22, 23, 25, 27, 28, 32], younger age [3, 15, 17, 27, 29, 32], poor economic status [13, 14, 25, 28], unemployment [14, 18, 25], and parental deprivation (broken home) [14, 15, 28, 30, 31], Other factors included having a lower education level [13, 28, 32], and peer-group influence [15, 22, 23, 28, 30, 31].

Younger population aged ≤35 years older was the most common group of people indulged in drug abuse based on the data of the included studies. The prevalence of drug abuse among this population may be the reason why some of the studies were specifically conducted among this group of people in the general population [11, 14], undergraduate students [17, 20, 26], and secondary school students [12, 16, 21–24, 26, 27, 30, 31]. Also, studies have shown that over 50% of persons with drug abuse-related psychiatric admission were secondary school students [29, 32]. These findings also suggest that the prevalence of drug abuse among secondary school students is high. Young people are the most valuable asset for sustainable social development in any society, but most of this population lacks awareness of substance addiction which would empower them to escape drug abuse. This limitation is demonstrated in a study carried out among secondary school teachers and students in Nigeria [37]. The study revealed that approximately 60% of students were never exposed to drug abuse education, while 73% of teachers reported that they currently did not teach their students about drug abuse education. This finding could be another reason for the highest prevalence in younger populations, and a crucial gap that could be targeted for interventions.

Determinants of drug abuse also included those related to family background. Individuals from polygamous family backgrounds, dysfunctional families (parental deprivation), being single and having parents or relatives who abuse drugs are more likely than those who are not to abuse drugs [14, 17, 18, 28]. Since in most cases children from broken families or polygamous families are left alone to feed themselves and their desire to go to school is often not of great concern to family members. Thus, to escape the reality of frustration and family strain, they find themselves entangled in substance abuse habits.

The peer-group influence was a predictor of drug abuse which most studies consistently reported. People with friends who abuse drugs are more likely than those with friends who do not abuse drugs to engage in the act [15, 22, 23, 30, 31].

The same author identified religion as a protective determinant of drug abuse in Nigeria in two studies [19, 20]. Makanjuola et al. found that undergraduate medical students who are religious are less likely than those who were not to engage in drug abuse [20]. Further, religious commercial drivers are less probable to engage in drug abuse than those who are not religious [19]. Commercial drivers in Nigeria constitute an integral part of socioeconomic growth, on which the majority of the public depend for transport. Preventing drug abuse among commercial vehicle operators in Nigeria will, therefore ensure optimum safety for the people.

### Psychiatric Admission due to Drug Abuse

Three studies were on psychiatric admission due to drug abuse [25, 29, 32]. The studies were conducted in Kaduna state (northwestern region) in 1986 [29], Edo state (south southern region) in 1976 [32], and the other was performed at all psychiatric facilities in Nigeria [25]. Findings of these studies showed the psychiatric symptoms resulting in admissions included toxic psychosis, anxiety state, schizophrenia, delusion [25, 32]. There was no suicide case recorded in all the studies.

### Policy and Laws Against Drug Abuse in Nigeria

In Nigeria, the fight against drug abuse is backed by federal laws as far back as 1935. Since then, many laws were enacted, while others were amended leading to the establishment of the National Drug Laws Enforcement Agency (NDLEA) -an agency with the sole responsibility of tackling drug abuse and related offences [38]. Some of the most important laws against the cultivation, trafficking, and abuse of illicit drugs in Nigeria are as follows [38]:

1. 1 The Dangerous Drugs Ordinance of 1935 enacted by the British Colonial administration.
2. 2 The Indian Hemp Decree No. 19 of 1966.
3. 3 The Indian Hemp (Amendment) Decree No. 34 of 1979.
4. 4 The Indian Hemp (Amendment) Decree, and the Special Tribunal (Miscellaneous Offences) Decree No. 20 of 1984.
5. 5 >The Special Tribunal (Miscellaneous Offences) (Amendment) Decree of 1986 and the National Drug Law Enforcement Agency Decree No. 48 of 1989 (as amended by Decree No.33 of 1990, Decree No 15 of 1992 and Decree No. 62 of 1999). These laws were harmonized as an Act of the parliament, CAP N30 Laws of the Federation of Nigeria (LFN) 2004. This Act established the NDLEA.

### Government Efforts in Preventing Drug Abuse in Nigeria

The NDLEA has been launching nationwide enforcement activities to seize drugs of abuse and arrest drug abusers in the community; sensitization program, rehabilitation and border patrol to checkmate trafficking of illicit drugs to and from Nigeria [38]. The 2019 NDLEA report has shown that in the last 10 years of operations, a total of 56, 745, 795, 555 kg of drugs were seized, 85, 058 persons with drug-related offences were arrested and 16, 937 cases were secured and convicted [39].

Recently, The Federal government of Nigeria, through Pharmacists Council of Nigeria (PCN) -an agency in charge of regulating the practice of pharmacy in Nigeria, banned the operation of open drug markets in Nigeria [40]. This effort was introduced to sanitize the drug distribution system in the country. The PCN also prohibits the handling of drugs by unlicensed personnel, especially prescription and controlled only drugs [41].

The National Agency for Foods and Drugs Administration and Control (NAFDAC), an agency of the Federal government of Nigeria, banned the manufacturing, importation and sale (without a valid prescription) of codeine and codeine-containing syrups in Nigeria [42]. In 2018, the agency shut down some pharmaceutical companies involved in the manufacturing of codeine-containing syrups in the country [43].

Other strategies by the Federal government include the establishment of the National Drug Control Master Plan (NDCMP) [44]. The NDCMP is a national blueprint for addressing the complex issues of drug trafficking, production, cultivation, and abuse in Nigeria. In 2018, the Federal government constituted a Presidential Advisory Committee for the Elimination of Drug abuse in Nigeria. The committee was saddled with the responsibility of advising the government on ways to reduce the increasing burden of drug abuse in the country [45]. However, the literature suggests that the burden of drug abuse may continue to rise in Nigeria due to the involvement of politics in law enforcement and lack of political goodwill [46, 47].

### Gaps Identified in the Included Literature

1. 1 Results from the included studies have shown that, despite the existence of Federal drug laws, and national drug policies and strategies, the burden of drug abuse and proliferation of controlled drugs are still on the increase in the country.
2. 2 Most of the epidemiological studies were conducted among secondary school students. Only a few studies were performed among the general population to identify other vulnerable groups of people involved in drug abuse.
3. 3 Only three studies were conducted on psychiatric admission related to drug abuse. Of the three studies, the most recent was published in 1986.
4. 4 Only a few studies reported sources of drugs, reasons for the abuse and risk factors associated with drug abuse. Identifying this information could guide the implementation of interventions.

### Recommendations

Reports from the included studies demonstrate that intervention measures to prevent drug abuse in Nigeria should be all-inclusive. The government, society, religious bodies, Non-Governmental Organisations as well as individuals all have a role to play. The efforts are numerous and not exhaustive. Below are some recommendations that might be applied to address the growing epidemic:

(1)The government should provide more employment opportunities to the youths and review existing drug laws to include stiffer penalties for offenders [38]. Allocation of funds to drug-related agencies should be increased to better the fight against the abuse of drugs. Enforcement activities should be more effective through strengthening the activities of taskforce at Federal and State governments levels. Nigerian borders should be well protected with surveillance activities by responsible agencies.
(2)Applying the conceptual model for understanding adverse drug events in the community [48]. Intervention measures based on Group-focused Cognitive Behavioral Health Education Program (GCBHEP) should be adopted to improve awareness and behavioral change [49]. Based on the model, other community-based education awareness in the form of mass campaigns through media houses both print and electronic, adverts, flyers, banners, radio jingles, lectures and other programs in religious places and public functions should be created and sustained [48].
(3)Secondary school-based programs aimed at encouraging healthy practices and lifestyle among adolescents would help to prevent substance use. There is also a need for periodic review of the curriculum in schools to introduce topics centered on dangers of drug abuse.
(4)Parents need to educate their children early enough on the risks associated with drug abuse [3, 15, 17, 27, 29, 32]. They should monitor the children closely and know the kinds of friends they are keeping [15, 22, 23, 28, 30, 31].

### Limitations

The present review has the following limitations; first, the systematic search of the literature was limited to two electronic databases. This approach may have excluded some eligible studies in the review. Secondly, we have not assessed the qualities of the included studies in this review. We have attempted to summarize their findings within this limitation and hope that readers, would be aware of such shortcomings, and be cautious in drawing conclusions from them.

## Conclusion

Findings from the epidemiological studies, reports and drug laws in Nigeria have shown that the burden of drug abuse is growing despite several drug laws, policies and strategic plans to prevent it. The prevalence is higher among the younger population, males, undergraduate and secondary school students, and commercial vehicle drivers. The most abused drugs included cannabis, amphetamine, codeine, cocaine and heroin. The major sources for the drugs were pharmacies/patent medicine stores, drug hawkers, friends who are abusers and drug pushers. The frequent reasons for indulging into drug abuse were to improve physical performance, to drive pleasure, desire to sleep, to experiment/curiosity motives, and to keep awake. Factors such as poor socioeconomic status, peer-group pressure, family problems and poor academic performance were commonly associated with drug abuse in Nigeria. Drug abuse has been a cause of many debilitating conditions such as schizophrenia and psychosis, leading to psychiatric admissions. Therefore, stringent measures and sustainable interventions are urgently needed to curb the increasing burden of drug abuse in Nigeria.

## Author Contributions

All authors listed have made a substantial, direct, and intellectual contribution to the work and approved it for publication.

## Conflict of Interest

The authors declare that the research was conducted in the absence of any commercial or financial relationships that could be construed as a potential conflict of interest.

## Abbreviations

AACODS, authority, accuracy, coverage, date, significance; FCT, federal capital territory; GCBHEP, group-focused cognitive behavioral health education program; FN, laws of the federation of nigeria; LSD, lysergic acid diethylamide; MeSH, medical sub-heading; NAFDAC, national agency for foods and drugs administration and control; NDCMP, national drug control master plan; NDLEA, national drug law enforcement agency; PCN, pharmacists council of Nigeria; PRISMA, preferred reporting items for systematic reviews and meta-analyses; UNODC, nations office on drugs and crime; YPS, young pharmacists scholars.

## Appendix 1

Search strategies used in the systematic search of the literature

**Table:**

Search	Query
A	Medline via Pubmed
#1	“Drug abuse” [MeSH terms]
#2	“Drug abuse” [title/Abstract]
#3	#1 OR #2
#4	“Drug, illicit” [MeSH terms]
#5	“Illicit drug use” [title/Abstract]
#6	#4 OR #5
#7	“psychoactive drugs” [MeSH terms]
#8	“psychoactive drugs” [title/Abstract]
#9	#7 OR #8
#10	#3 AND #6 AND #9
B	Google scholar
#1	“Drug abuse, illicit drug abuse, psychotropic abuse in Nigeria”
